# Circuit Techniques to Improve Low-Light Characteristics and High-Accuracy Evaluation System for CMOS Image Sensor

**DOI:** 10.3390/s22166040

**Published:** 2022-08-12

**Authors:** Norihito Kato, Fukashi Morishita, Satoshi Okubo, Masao Ito

**Affiliations:** Renesas Electronics Corporation, Kodaira 187-8588, Japan

**Keywords:** CMOS image sensor, column-parallel ADC, multi-functional fine pattern generator, on-chip test

## Abstract

The surveillance cameras we focus on target the volume zone, and area reduction is a top priority. However, by simplifying the ADC comparator, we face a new RUSH current issue, for which we propose a circuit solution. This paper proposes two novel techniques of column-ADC for surveillance cameras to improve low-light characteristics. RUSH current compensation reduces transient current consumption fluctuations during AD conversion and utilizing timing shift ADCs decreases the number of simultaneously operating ADCs. These proposed techniques improve low-light characteristics because they reduce the operating noise of the circuit. In order to support small signal measurement, this paper also proposes a high-accuracy evaluation system that can measure both small optical/electrical signals in low-light circumstances. To demonstrate these proposals, test chips were fabricated using a 55 nm CIS process and their optical/electrical characteristics were measured. As a result, low-light linearity as optical characteristics were reduced by 63% and column interference (RUSH current) as an electrical characteristic was also reduced by 50%. As for the high-accuracy evaluation system, we confirmed that the inter-sample variation of column interference was 0.05 LSB. This ADC achieved a figure-of-merit (FoM) of 0.32 e-·pJ/step, demonstrating its usefulness for other ADC architectures while using a single-slope-based simple configuration.

## 1. Introduction

CMOS image sensors are widely used in so-called digital still cameras such as smartphones, compact cameras, and single-lens reflex cameras, as well as in surveillance cameras, industrial applications [1,2,3], object recognition [4,5,6,7,8], ToF sensors for distance measurement [9,10,11], and medical applications [12,13,14]. The performance requirements in these fields are low noise, high speed, wide dynamic range, and high resolution, and various technologies have been reported to improve these performances. In particular, from the perspective of surveillance and security, improved low-light characteristics that enable clear images even in dark environments have become an important technology for image sensors in recent years. In order to process signals from pixels at high speed, such image sensor devices incorporate thousands of ADCs in the chip to enable high-speed digital output; of the available ADCs, pixel ADCs [15,16,17,18] and column ADCs [19,20,21,22,23,24,25,26,27,28,29] have been used as a way to achieve widely parallel operation. To achieve high resolution and low power consumption, a column ADC is the best choice, because a pixel ADC is disadvantageous in terms of power, heat, and mounting.

There are several types of ADCs suitable for column-parallel operation, including single-slope [19,20,21,22,23,24,25,26], successive approximation register (SAR) [27], cyclic [28], delta-sigma [29], and folding integration [30], which provide optimal performance for each configuration. These ADCs are used in different ways depending on the application: single-slope ADCs are suitable for commercialized CMOS image sensors with compact size, while SAR and cyclic ADCs with binary search capability are suitable for high-speed applications. Delta-sigma ADCs are also suitable for high-speed, high-resolution applications. In addition, folding integration ADCs are used for low-noise applications.

**Figure 1:**
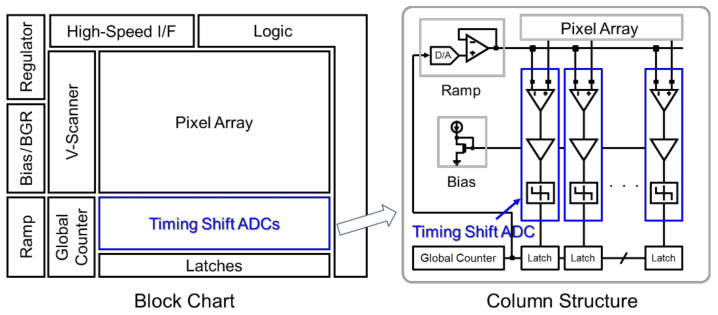
Block chart of an image sensor and one column structure.

Figure 1 shows a schematic diagram of the image sensor we adopted. In this study, we focus on applications for surveillance cameras that can capture images even in low-light conditions. Since the surveillance cameras are targeted at the volume zone, the configuration is designed with the highest priority being area reduction for the purposes of cost reduction. Originally, our prior ADC comparator used a two-stage full differential amplifier [23]. However, since the full differential amplifier had a large number of transistors and capacitance elements, we adopted a simple scheme in which the second stage was a single-ended amplifier in order to achieve a smaller ADC. Such a single-ended scheme itself has already been reported [22]. However, the RUSH current issue, as mentioned in Section 2, causes linearity characteristic degradation especially in low-light applications, so we devised a way to solve this problem while still achieving a compact ADC size.

In single-slope ADC, since many ADCs perform AD conversion simultaneously, there is concern about the increase in circuit noise due to their simultaneous operation. Especially in the case of surveillance cameras, since the characteristics under low illumination with few optical signals are important, the column ADCs need to amplify weak electrical signals and are relatively susceptible to noise due to circuit operation. To solve this problem, we propose a current compensation circuit and a timing shift ADC to reduce the number of simultaneous ADC operations [7]. In column ADCs, the power supply GND noise is highest during the reset operation, when all the thousands of ADCs operate simultaneously, because all the comparators that make up the ADC circuit operate simultaneously, causing a large voltage drop. Timing-shift ADCs can reduce the number of ADCs operating simultaneously by shifting the ADC operation time for each column, thereby improving voltage drop and preventing the degradation of pixel characteristics in dark mode.

We actually created a chip equipped with this ADC and compared its performance against the conventional method to demonstrate that low-light characteristics can be greatly improved. In the demonstration, since the number of signals handled by the ADC itself was very small, the effects caused by such simultaneous operation were observed through actual electrical measurement, and an evaluation device for measuring electrical characteristics was also introduced. Beginning with the fact that an electrical testing method is necessary to observe the operation of an ADC with an optical input image sensor, we solved this problem by providing an on-chip dual-path test circuit [31] that electrically measured the ADC by keeping actual CIS operating conditions. By using this technique, highly accurate measurement of the ADC performance was realized without the influence of optical input or pixel characteristics. This dual-path test circuit was capable of measuring not only INL and DNL, but also various electrical characteristics such as rush (peak) current characteristics due to simultaneous ADC operation, adjacent crosstalk interference, and horizontal smear characteristics.

Furthermore, in order to measure weak electrical signals, a high-precision evaluation device was necessary to minimize the influence of noise and disturbance noise from the evaluation environment. We analyze the measurement results by comparing the optical input measurement and the electrical signal measurement and demonstrate that the proposed timing shift ADC improves the low-light characteristics of the image sensor itself as well as granting a performance improvement as an ADC.

In Section 2, we explain the design issues of conventional column ADCs and propose a current compensation circuit and a timing shift ADC as those countermeasures. In Section 3, we present an evaluation environment for measuring low-light characteristics. In Section 4, high accuracy and low noise evaluation results are demonstrated through the measurement of various electrical characteristics and the results of test chip measurements. Finally, we conclude in Section 5.

## 2. Design Issues of Conventional Column ADCs and Corresponding Countermeasures

**Figure 2:**
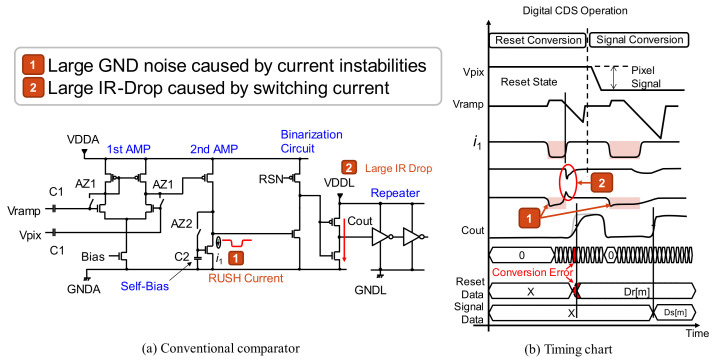
ADC comparator of conventional scheme.

Figure 1 shows the block chart of our image sensor and one column structure. In this scheme, timing shift ADCs are used to improve low-light characteristics. To clarify the design issues of simultaneous ADC operation, Figure 2 describes ADC operation with a conventional comparator and its timing chart. In the ADC operation, we used a digital CDS (correlated double sampling) scheme in which the circuit offset is canceled by executing reset conversion and signal conversion. In the column-wise ADC structure, all ADCs operate at the same time during the reset conversion. Therefore, the supply voltage fluctuation deteriorates the conversion characteristics. The first issue is large GND noise caused by current instabilities. ADC current instabilities due to the state change of comparators induce an analog GND fluctuation; this is called the RUSH current during the ADC operation. The second issue is a large IR-drop caused by the repeater logic current switching. Through current between the ADC comparator and the repeater causes a large IR-drop of the logic voltage VDDL and its ground GNDL. These instabilities cause conversion error within the ADC, and deteriorate low-light linearity characteristics in particular because the output code itself becomes small in low-light.

**Figure 3:**
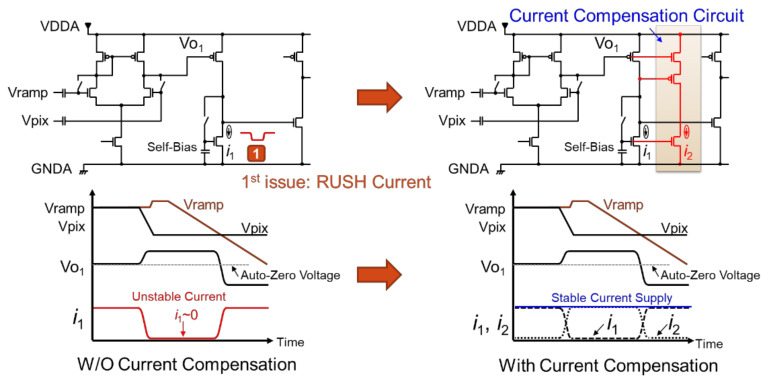
RUSH current compensation.

We will now discuss the first issue of RUSH current due to unstable current of the amplifier. Figure 3 shows the current mechanism of RUSH and its compensation circuit. In the configuration without current compensation, the current $i_{1}$ in the second-stage amplifier changes depending on the output state of $Vo_{1}$, which is the output voltage of the first-stage amplifier. This unstable current fluctuates the analog ground *GNDA*, leading to an error during AD conversion. To suppress the fluctuation of this $i_{1}$ current, in the configuration with current compensation, the $i_{2}$ current path is added to compensate for current in the complementary. In this scheme, the supply current of the second-stage amplifier $\left(i_{1}+i_{2}\right)$ becomes constant.

**Figure 4:**
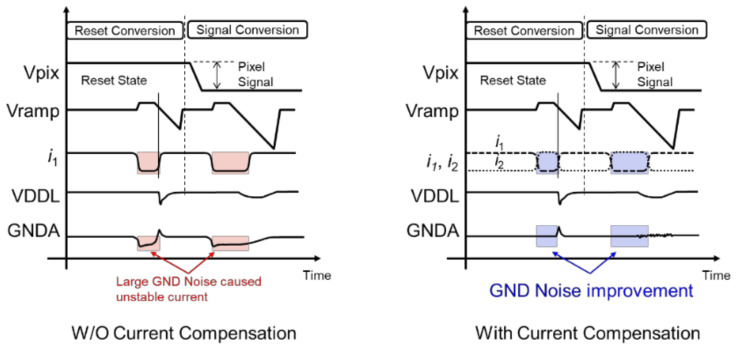
The impact of RUSH current compensation.

The impact of RUSH current compensation is shown in Figure 4. Without current compensation, by changing $i_{1}$, the GND fluctuations listed in the reset conversion and the signal conversion induce large GND noise during each operation period. On the other hand, in the configuration with current compensation, the amplifier current $\left(i_{1}+i_{2}\right)$ can be kept stable, and GND noise due to signal transition can be greatly reduced.

**Figure 5:**
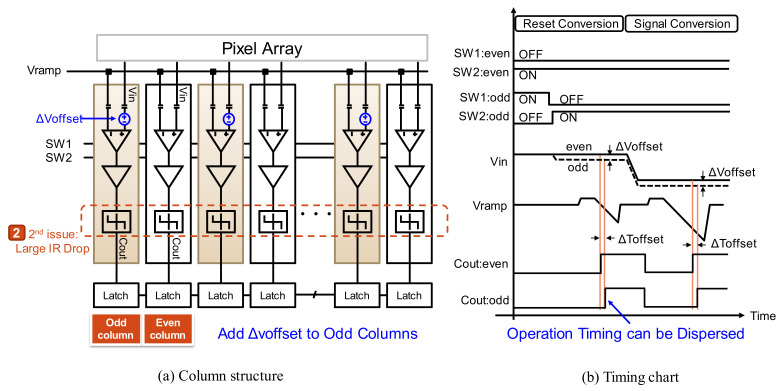
Concept of timing shift ADC.

Figure 5 shows the concept of timing shift ADC, which can reduce large IR drop by means of simultaneous ADC operation—our second issue. In our column ADC structure, the behavior of even and odd columns is differentiated and the offset voltage Δ*Voffset* is generated only by the odd columns. For single-slope ADCs, this voltage offset is the difference in conversion time Δ*Toffset*. Therefore, the comparator’s output timing Cout_even of even columns and Cout_odd of odd columns can be shifted, and as a result, the simultaneous operation of ADCs can be reduced by half.

**Figure 6:**
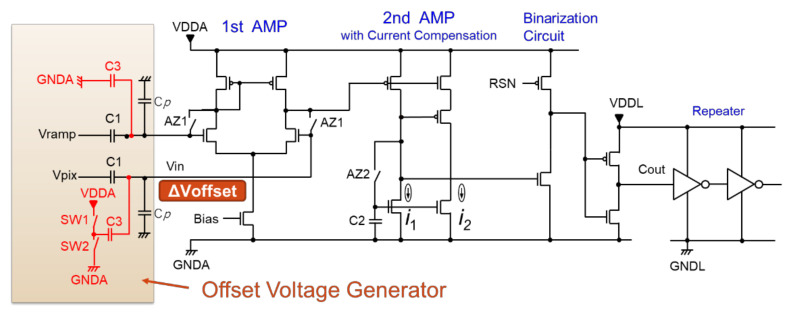
Circuit diagram of timing shift ADC.

A circuit diagram of the timing shift ADC is shown in Figure 6. To add the voltage offset to the input signal $V_{in}$, we provide capacitance *C*3 and switches SW1 and SW2. By switching SW1 and SW2 on and off, the left-side voltage of *C*3 can be changed from *VDDA* to *GNDA*. Here, *Cp* is the parasitic capacitance of the first-stage amplifier input.

**Figure 7:**
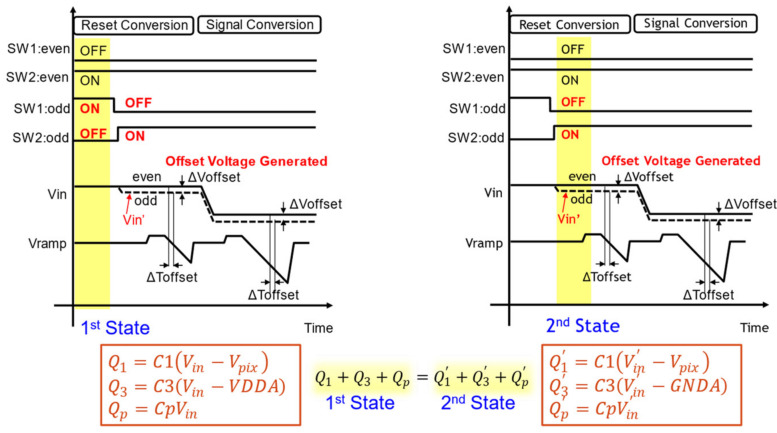
Generation of offset voltage.

Figure 7 shows how to attach the offset voltage. This figure describes the behavior of odd columns on the offset side. When the reset conversion of digital CDS starts, the state of each switch SW1 and SW2 is as described in the first state period of Figure 7. In this state, SW1 is ON and SW2 is OFF, and the electric charge stored in each capacitance at this time is shown in Equation (1).

(1) $$Q_{1}=C1\left(V_{in}-V_{pix}\right),Q_{3}=C3\left(V_{in}-VDDA\right),Q_{p}=CpV_{in}$$

From this state, we change the SW to add the offset to the Vin node of the ADC input. This is the second state. In the second state, SW1 is turned off and SW2 is turned on. Then, the voltage of the input Hi-Z node Vin changes from $V_{in}$ to $V_{in}^{’}$, and the charge stored in those capacitors also changes, as shown in Equation (2).

(2) $$Q_{1}^{’}=C1\left(V_{in}^{’}-V_{pix}\right),Q_{3}^{’}=C3\left(V_{in}^{’}-GNDA\right),Q_{p}^{’}=CpV_{in}^{’}$$

As the charge conservation law between the first state and the second state is established, the offset voltage can be expressed in Equation (3).

(3) $$\Delta Voffset=V_{in}-V_{in}^{’}=\frac{C3}{C1+C3+Cp}\left(VDDA-GNDA\right)$$

Therefore, when compared to even columns, it is possible to create a time difference of Δ*Toffset* caused by this Δ*Voffset*, and as a result, we can avoid the same-time operation of all ADCs.

Although this Δ*Voffset* has the effect of shifting the AD conversion time by Δ*Toffset*, it does not adversely affect the AD conversion characteristics after digital CDS, even if there are differences between even–odd columns or mismatched capacitance values for each column, because the term in Equation (3) itself is cancelled by the same value as the difference between the signal and reset AD conversion results through digital CDS, as described in Figure 2.

**Figure 8:**
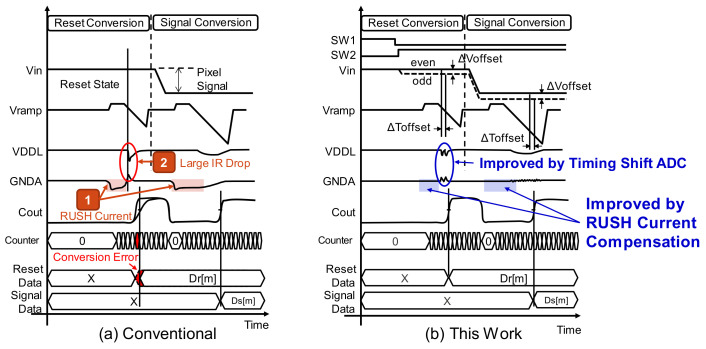
Improvement image by RUSH current compensation and timing shift ADC.

Figure 8 shows the timing diagram of the conventional scheme and the proposed one. This exhibits the two issues mentioned so far and their corresponding improvements under the proposed scheme. The RUSH current is improved by the current compensation circuit, and the large IR drop of the logic voltage is improved by the timing shift ADC, enabling stable AD conversion without worrying about power supply noise.

## 3. Evaluation Method

The circuits proposed in the previous section have lower simultaneous operating noise than conventional circuits. Therefore, an evaluation system capable of detecting operating noise with high accuracy was required. This being said, even if the evaluation system can achieve low noise, if the evaluation results are not reproducible, the evaluation must be repeated many times to check the validity of the measurement results, which causes evaluation to take an enormous amount of time. In this section, we first describe the common evaluation method of image sensors and its issues, and then propose a high-precision evaluation system that solves the issues.

### 3.1. Common Evaluation Method of Image Sensors and Evaluation Issues

The most common method of evaluation for image sensors is using optical input. Since general image sensors have linear input–output characteristics with respect to light intensity, sensor linearity is evaluated based on the difference between the ideal input/output characteristics and the characteristics of the actual measurement results. However, this evaluation method using optical input has been reported to have the following issues [31]: The first issue is that the pixel and ADC characteristics cannot be evaluated separately. If separate evaluation is not possible, it is impossible to directly confirm whether the problem is on the pixel or the ADC side. The second issue is that it is extremely difficult to accurately expose the inter-column interference noise measurement pattern to the image sensor surface due to light diffraction. To solve these problems, a method of measuring only the electrical characteristics of the ADC by providing a direct electrical signal input path to the ADCs has been proposed [31]. However, although previous work has reported that it is possible to measure with high precision, the issue remains that it has not been confirmed whether the measurement results are reproducible.

### 3.2. Proposed Evaluation System

**Figure 9:**
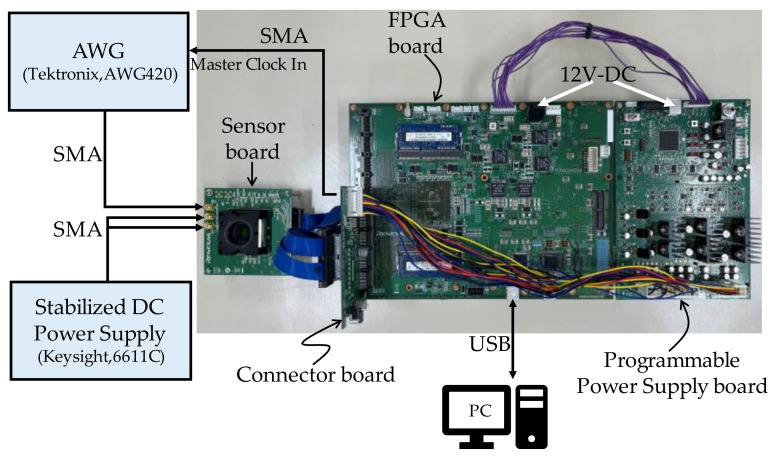
Evaluation system.

Applying previous work to confirm reproducible and accurate measurements [31], we constructed the evaluation system shown in Figure 9. The evaluation system consisted of two measurement instruments, four evaluation boards, and a PC. The instruments were used as input signals for evaluating the electrical characteristics of the ADC. The evaluation boards consisted of an image sensor board, a connector board, an FPGA board, and a programmable power supply board.

**Figure 10:**
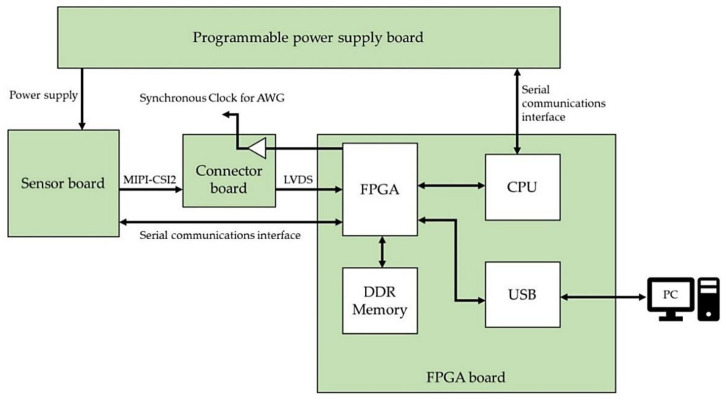
System overview.

The operation of this system from start to finish of the evaluation is described in Figure 10. First, the power supply voltage settings and image sensor register data are read from the PC via USB. Next, the setting data read from the PC is stored in the field-programmable gate array (FPGA), and only the power supply setting data for the image sensor is passed to the central processing unit (CPU), which in turn sends the power supply voltage information to the programmable power supply board via the serial communications interface. The programmable power board executes the power-up sequence for the image sensor based on the received information. Then, register data for the image sensor is sent from the FPGA to the image sensor via the serial communications interface to write the initial settings to the image sensor. After the initial settings are completed, the image sensor is shifted into the operation mode to get data for optical evaluation or ADC electrical characterization. Digital data, which are the evaluation data output from the image sensor, are sent to the FPGA via the connector board. The FPGA accumulates the digital data on double data rate (DDR) memory, generates a RAW image when data accumulation for one frame is completed, and transfers the RAW image to the PC via USB.

During the operation mode period, the FPGA outputs synchronization clocks for the arbitrary waveform generator (AWG) and the image sensor, and the AWG output timing and the analog-to-digital (AD) conversion timing of the image sensor are synchronized. When the measurement data acquisition is completed, the programmable power supply board executes a power-down sequence to terminate the evaluation. The acquisition data of this evaluation system are RAW images for both optical evaluation and electrical characteristic evaluation. This brings the operating state of the image sensor during image acquisition, which is the normal operation of the image sensor, and the operating state during ADC electrical characteristic evaluation closer, and makes it easier to reproduce the interference noise caused by ADC operation during the evaluation of electrical characteristics. Furthermore, since measurement data from all the several thousand image sensor ADCs can be obtained at once, if any ADCs are affected by operating noise, this can be easily confirmed because the result will be different from that of unaffected ADC outputs.

**Figure 11:**
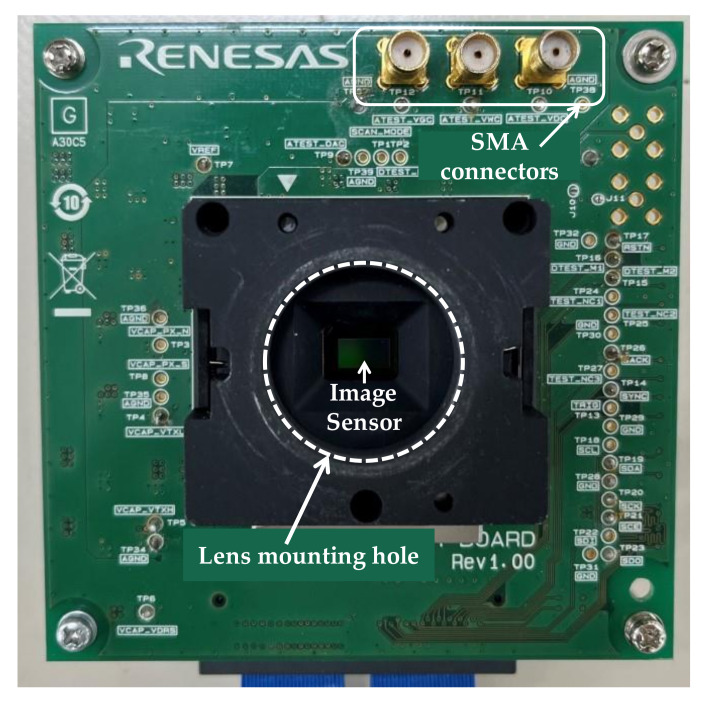
Sensor board.

The roles of each board are described below: The sensor board attaches the image sensor to be evaluated and provides access to input and output signals to the image sensor. This board is designed for both optical and electrical characterization. As shown in Figure 11, the socket of the sensor board has a hole for lens mounting, and a lens for optical evaluation can be attached there. On the other hand, in the case of evaluating the electrical characteristics of the ADC, the socket hole is plugged with a cap to prevent light from entering the image sensor, and the evaluation is performed using electrical signals from the measurement instruments connected with sub-miniature type A (SMA) cables.

**Figure 12:**
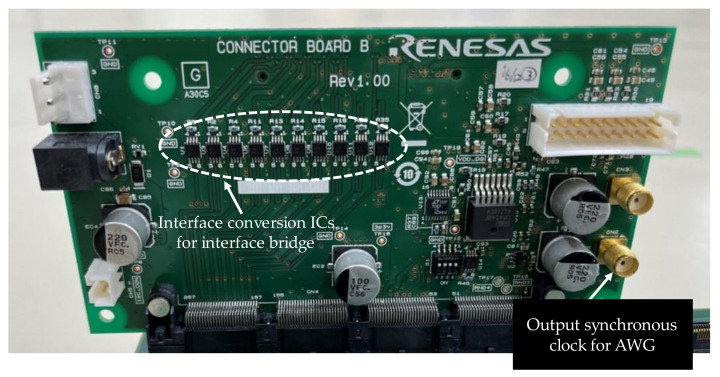
Connector board.

The connector board functions as an interface bridge as shown in Figure 12. Even if the sensor board and FPGA board use different high-speed interface standards, this connector board outputs signals that match the interface standard of the FPGA board. It also has the function of outputting a synchronization clock generated by the FPGA to the AWG.

**Figure 13:**
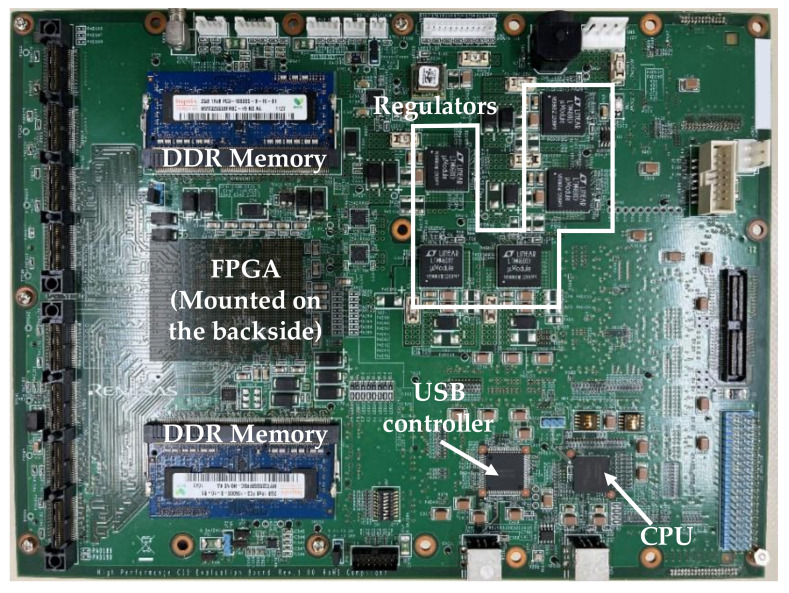
FPGA board.

The FPGA board shown in Figure 13 has three roles: The first is to create a RAW image from the digital data of the sensor output and then transfer the RAW image from the FPGA board to the PC via the USB interface. The second is to control the register settings of the image sensor via serial communication. The third role is to generate synchronization clocks for the image sensor and the AWG.

**Figure 14:**
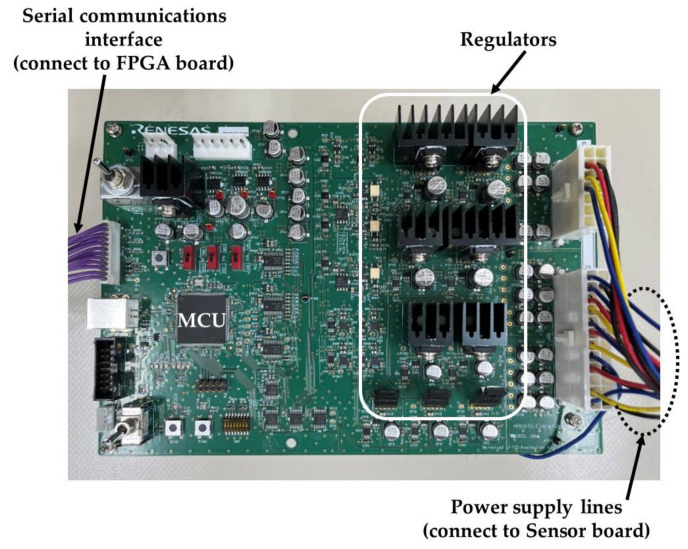
Programmable power supply board.

The programmable power supply board shown in Figure 14 generates the power supply voltage supplied to the image sensor. The generated voltage can be controlled on the order of millivolts. It is also possible to control the power-on and power-off of the supply power node on the order of milliseconds, allowing the power-up and power-down sequences of the image sensor to be executed. Devices such as DC power supplies themselves can be noise sources and can be a factor in increasing environmental noise. Therefore, it is better to minimize their use. We used the programmable power supply board to reduce environmental noise. To relax physical placement restrictions between the sensor board and the programmable power supply board, the regular cables were used for the power supply lines, but ferrite beads and decoupling capacitors were placed on the back side of the sensor board to reduce power supply noise.

## 4. Measurement Results

**Figure 15:**
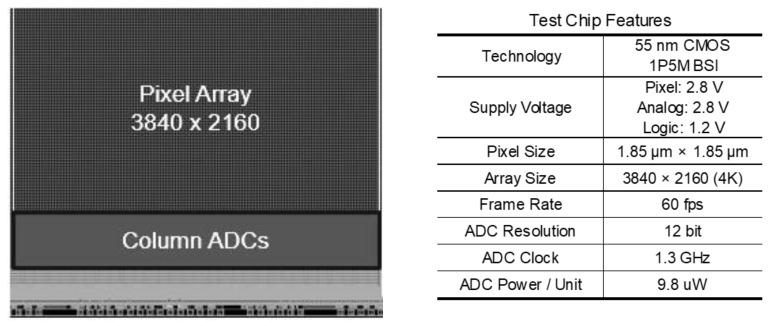
Test chip features.

The test chip features are shown in Figure 15. We fabricated the test chips using a 55 nm CIS process. The image sensor performance is capable of capturing video at 4 K/60 fps. The ADC resolution is 12-bit and the ADC power consumption per unit is 9.8μW, achieving low power consumption.

Table 1 shows the electrical characteristics evaluation results of three test samples in the range of power supply voltage of ±5% and temperatures of −25 °C, 27 °C, and 90 °C. Each test sample had 3840 column-parallel ADCs implemented, and the results in Table 1 show the best and worst values of the experimental results for all ADCs under all measurement conditions. The worst results of differential non-linearity (DNL) and integral non-linearity (INL) were 0.55 LSB (Sample 2) and 4.81 LSB (Sample 1), respectively, and the variability among three samples were 0.08 LSB and 2.65 LSB, respectively. Furthermore, the result of this accelerated column interference was 2.5 LSB, whereas the result for the conventional method was 5.0 LSB [7]. Therefore, the proposed technique reduced column interference by 50%. As for the accelerated column interference, the variability among three samples was 0.05 LSB.

**Figure 16:**
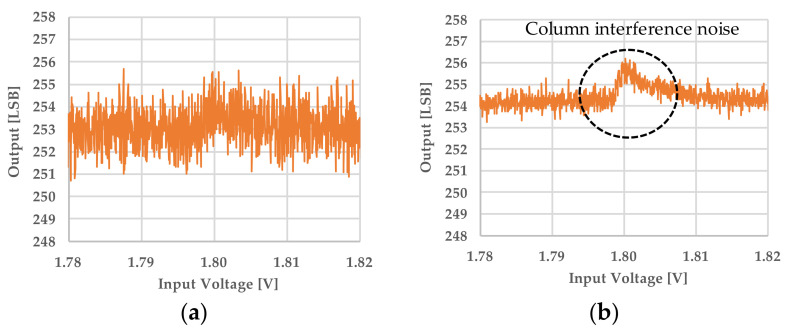
Comparison of accelerated column interference evaluation results between the conventional and the proposed evaluation system. (**a**) shows the measurement result using the conventional evaluation system and (**b**) shows the result using the proposed evaluation system.

Figure 16 shows a comparison of acceleration column interference evaluation results between the conventional and proposed evaluation systems. The measurement results of the conventional system are noisy and the points where interference noise occurs are unclear. In contrast, the proposed system measures with lower noise, and the point where the interference noise occurs is clear.

**Figure 17:**
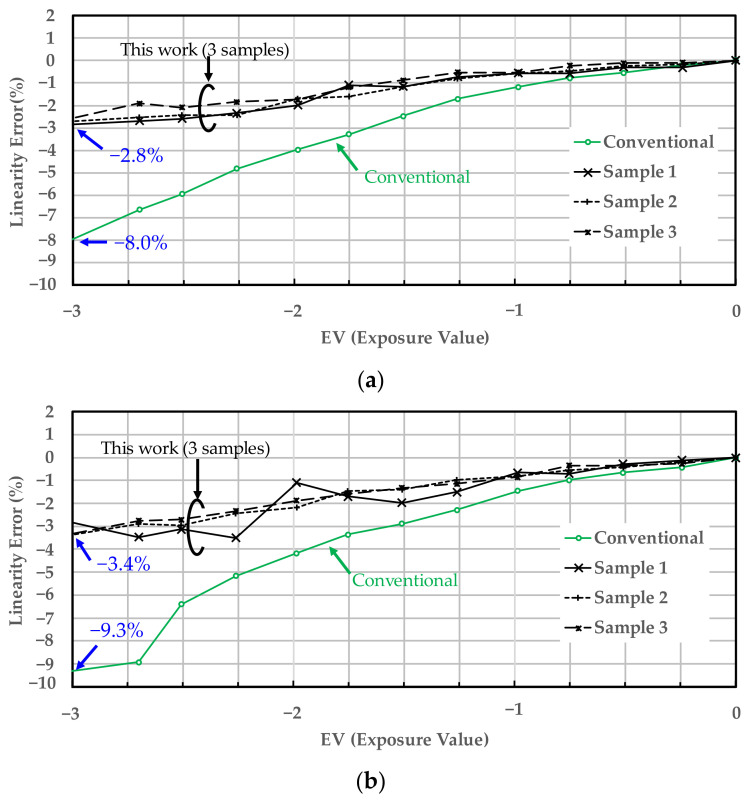

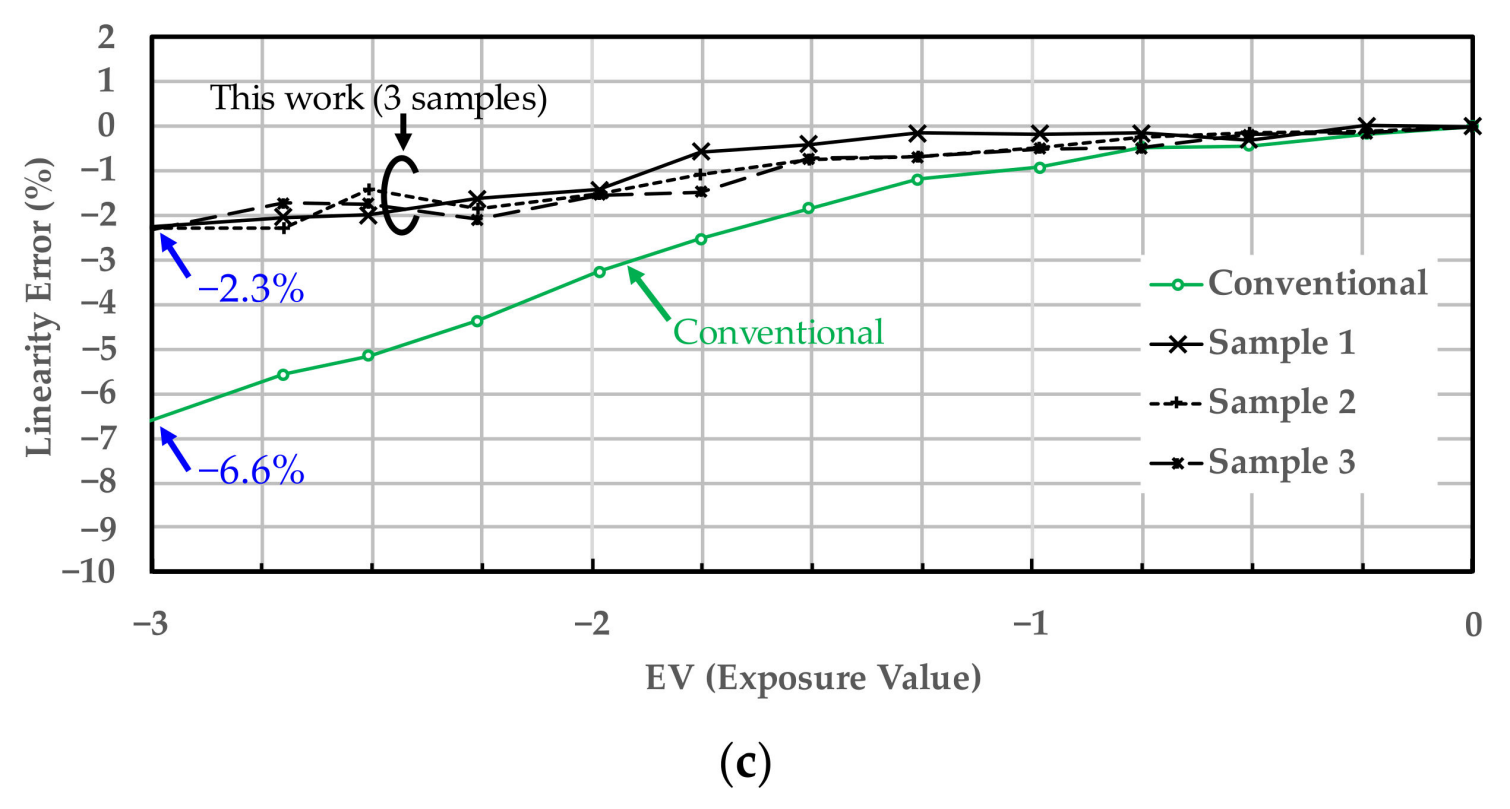
Comparison of sensor linearity error under low-light conditions between the conventional and proposed circuit. (**a**–**c**) show the evaluation result at typical, minimum, and maximum operating voltage, respectively.

Next, we confirmed the improvement effect of the proposed circuit to reduce simultaneous operation noise. In low-light environments, linearity is degraded due to weak signals, which are recognized as vertical stripes in the image. Moreover, in low-light conditions, optical shot noise is low and column ADC operation noise is easily seen as vertical stripe noise (VSN) for each column. Therefore, we evaluated two items, sensor linearity under low-light conditions and VSN under low-light conditions, using three sample test chips fabricated on a 55 nm CIS process. We measured the sensor linearity within a supply voltage range of ±5% and at room temperature only. Figure 17a–c show the results of sensor linearity evaluation under low-light conditions. The proposed circuit improved the sensor linearity error to −3.4% compared to −9.3%, the worst result of the conventional circuit. We confirmed that the sensor linearity error was reduced by 63% even if the improvement effect of this work was the smallest.

**Figure 18:**
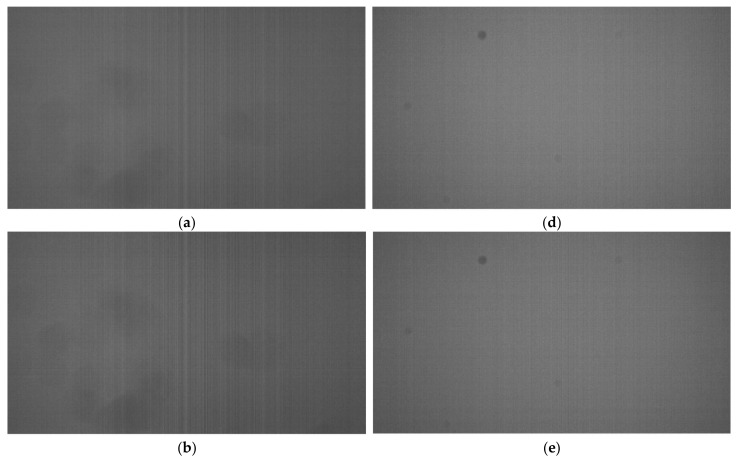

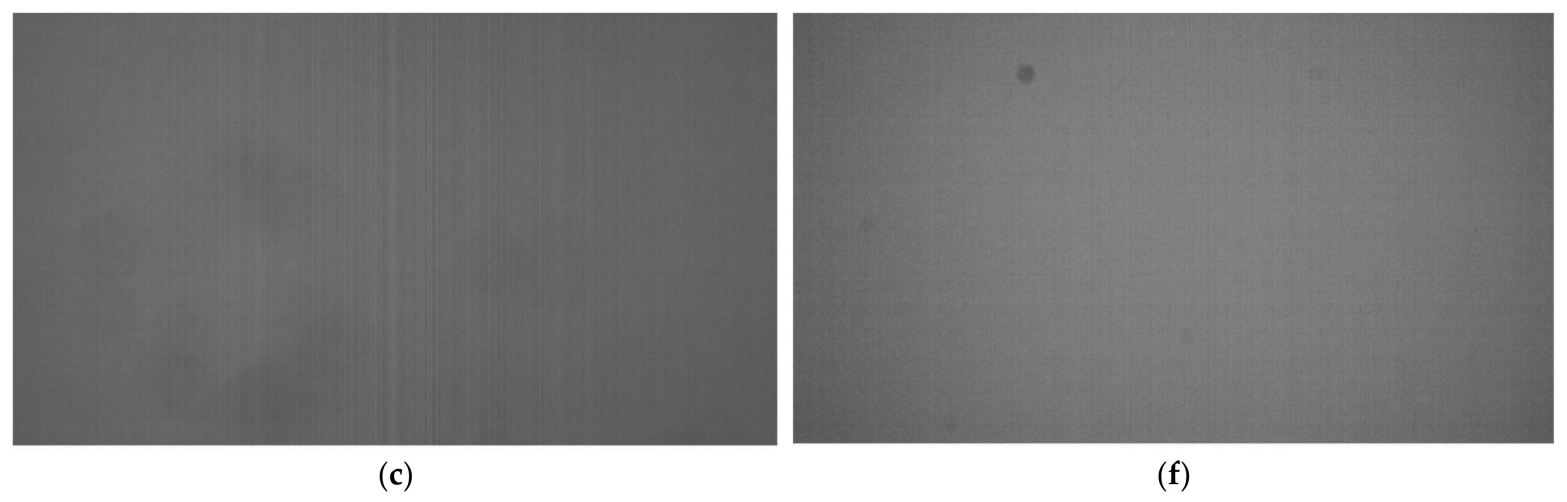
(**a**–**c**) are images taken under a low-light condition using conventional circuit. The operating voltages are as follows: (**a**) Typical operating voltage, (**b**) minimum operating voltage, and (**c**) maximum operating voltage. (**d**–**f**) are images taken with the proposed circuit under the same illumination as (**a**–**c**). The operating voltages are as follows: (**d**) typical operating voltage, (**e**) minimum operating voltage, and (**f**) maximum operating voltage. All images were enhanced with 384x digital gain.

The results of VSN evaluation under low-light conditions are shown in Figure 18. In this figure, an image is an average of 10 RAW images. This averaging process reduces random noise, resulting in an image in which VSN is more emphasized. This VSN is not observed in complete darkness, but only in low-light conditions. On the other hand, it becomes invisible under bright conditions because the shot noise of the photodiode increases. In the image of the conventional circuit, the vertical stripes are clearly visible near the center of the image, but in the image of the proposed circuit, the vertical stripes are almost invisible. The cause of this VSN is also the AD conversion error caused by the simultaneous operation noise as well as the sensor linearity, and since the proposed circuit can reduce the simultaneous operation noise, the AD conversion error is also reduced; as a result, the vertical stripe is no longer visible.

**Figure 19:**
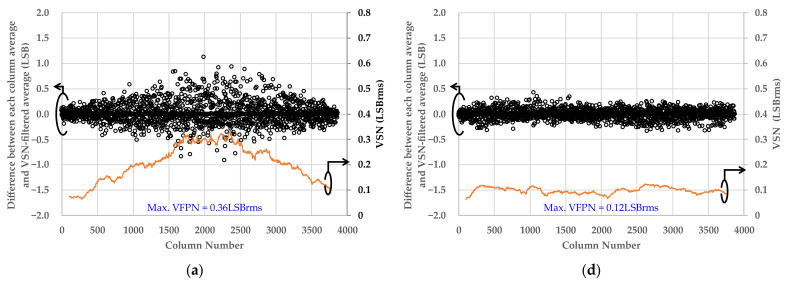

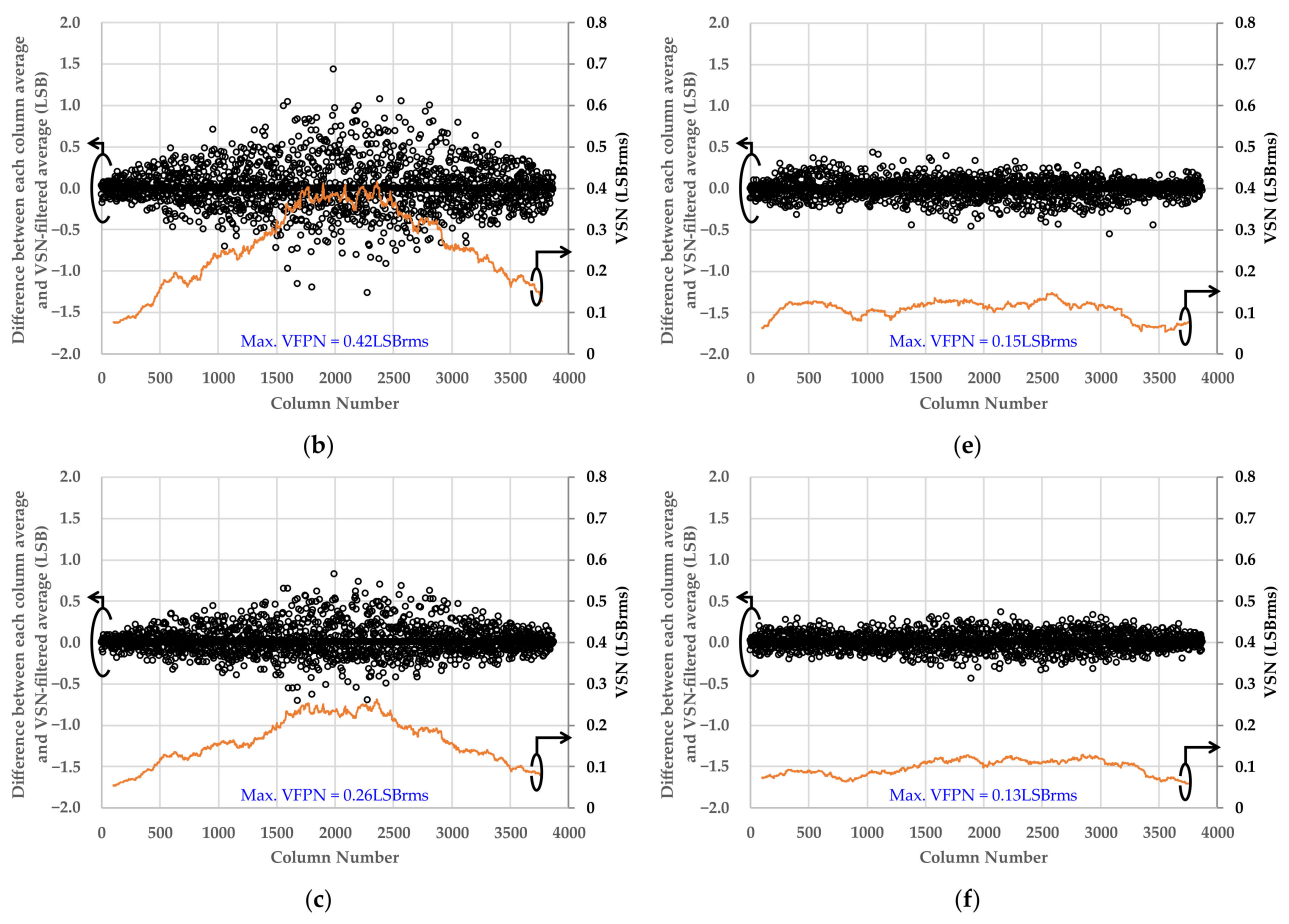
(**a**–**c**) show the VSN under the low-light condition calculated from images in Figure 18a–c taken with the conventional circuit. The operating voltages are as follows: (**a**) Typical operating voltage, (**b**) minimum operating voltage, and (**c**) maximum operating voltage, respectively. (**d**–**f**) show the VSN under the low-light condition calculated from the Figure 18d–f taken by the proposed circuit. The operating voltages are as follows: (**d**) typical operating voltage, (**e**) minimum operating voltage, and (**f**) maximum operating voltage, respectively. All graph data are values of digital gain ×1.

Figure 19 shows the difference between the average value of each column in Figure 18 and the average value filtered for the VSN component and its variations. The VSN filtered average was calculated by applying a median filter to each column average. The VSN was calculated from the variance of the difference for every 100 columns. The condition of maximum improvement was at the typical operating voltage in Figure 19, and the Max. VSNs of the conventional and proposed scheme were 0.36 LSB and 0.12 LSB, respectively. In this case, VSN was improved by 67%. On the other hand, the condition of minimum improvement effect was at the maximum operating voltage in Figure 19, where the Max. VSNs of the conventional and proposed scheme were 0.26 LSB and 0.13 LSB, respectively. In this case, VSN was improved by 50%. This was worst case, and we confirmed that the proposed scheme improved VSN by at least 50%. Finally, Table 2 summarizes the performance of the implemented ADC compared with prior works with FoM [e-·pJ/step].

## 5. Conclusions

We proposed circuit techniques, RUSH current compensation, and timing shift ADC to realize a high-sensitivity image sensor. The proposed circuits reduced AD conversion errors due to simultaneous operating noise and decreased sensor linearity degradation even under low-light conditions with few optical signals. A lower-noise evaluation system was needed to measure our high-sensitivity image sensor; since measurement instruments such as DC power supplies can also be noise sources, the proposed evaluation system was configured with the minimum number of measurement instruments. To confirm these proposals, test chips were fabricated using a 55 nm CIS process and the low-light characteristics were measured. The proposed technique improved the low-light linearity error and column interference noise (RUSH current) by 63% and 50%, respectively. As for the high-accuracy evaluation system, we confirmed that the inter-sample variation of column interference was 0.05 LSB.

## Figures and Tables

**Table 1:** ADC electrical characteristics evaluation results.

Measurement Items	Sample 1 (Best/Worst)	Sample 2 (Best/Worst)	Sample 3 (Best/Worst)
Random noise [LSBrms]	0.85/0.98	0.83/0.96	0.85/0.96
Fixed pattern noise [LSB]	0.28/0.36	0.28/0.36	0.28/0.37
Min INL [LSB]	−1.27/−1.86	−1.72/−2.07	−1.55/−1.91
Max INL [LSB]	2.09/4.81	1.09/2.16	1.38/2.41
Min DNL [LSB]	−0.35/−0.43	−0.34/−0.44	−0.36/−0.41
Max DNL [LSB]	0.38/0.51	0.39/0.55	0.46/0.53
Min adjacent column INL difference [LSB]	−0.92/−1.25	−0.91/−1.23	−0.91/−1.17
Max adjacent column INL difference [LSB]	0.96/1.20	0.93/1.18	0.96/1.14
Absolute gain error [dB]	0.27/−0.70	0.22/−0.75	0.41/−0.61
Cross talk [LSB]	0.38/0.61	0.45/0.53	0.38/0.50
Accelerated column interference [LSB]	2.31/2.48	2.30/2.45	2.34/2.50

**Table 2:** Performance comparison.

	Unit	This Work	[24] Sensors 2020	[32] ISSCC 2016	[30] JSSC 2012	[33] TCAS-I 2019	[34] JSSC 2022	[35] JSSC 2019
Process Technology	−	55 nm 1P5M BSI	90 nm	45 nm 1P4M/ 65 nm 1P5M	180 nm 1P4M	130 nm 1P3M FSI	65 nm	90 nm
Power supply	V	2.8 (pixel, analog)/1.2 (digital)	2.8 (analog)/ 1.5 (digital)	2.5, 2.8 (analog)/ 1.2, 2.5 (digital)	3.3 (analog)/ 1.8, 3.3 (digital)	3.3 (analog)/ 1.5 (digital)	2.8 (analog)/ 1.05 (digital)	−
Pixel size	um^2	1.85 × 1.85	−	1.1 × 1.1	7.5 × 7.5	5.6 × 5.6	4.95 × 4.95	2.8 × 2.8
Pixel array (H × V)	pixels	3840 × 2160	960 × 720	7728 × 4368	1032 × 1024	1024 × 128	1668 × 1364	1232 × 952
Frame rate	fps	60	35	240	2.2 @ 128smpls.	−	30/1200	75
Power consumption	W	0.3	0.03	3	0.45	0.02	0.12/0.60	−
ADC architecture	−	Single Slope	2−step Single Slope	3−stage cyclic based	Folding integration cyclic	Flash TDC− interpolated	Single Slope	SAR
ADC resolution	bit	12	12	12	13–19	12	10	10.7
ADC power consumption	uW/column	9.8	6.35	120	−	177	23.9	2.1
ADC DNL	LSB @ 12 bit	+0.55/−0.44 @wst (*1)	+4.25/−1.00	+0.82/−0.88	−	+1.1/−0.4	−	+0.39/−0.36
ADC INL	LSB @ 12 bit	+4.81/−2.07 @wst (*1)	+5.73/−7.30	+1.04/−11.75	−	+5.8/−8.2	−	+2.31/−0.79
FPN	uVrms	87 @wst (*1)	−	−	36	−	−	29
Random noise	uVrms	273	472	414	65 @ 128smpls.	477	294	407.75
FoM (*2)	e-·pJ/step	0.32	0.56	0.33	0.35	−	0.52	−

(*1) wst: worst value of power supply ±5%, temperature −25°C to 90°C, and 3 samples; (*2) FoM: (power × noise)/(pixels × fps × 2^bit).

## Data Availability

Not applicable.
